# Presence of a 34-gene signature is a favorable prognostic marker in squamous non-small cell lung carcinoma

**DOI:** 10.1186/s12967-020-02436-3

**Published:** 2020-07-03

**Authors:** W. S. M. E. Theelen, O. Krijgsman, K. Monkhorst, T. Kuilman, D. D. G. C. Peters, S. Cornelissen, M. A. Ligtenberg, S. M. Willems, J. L. G. Blaauwgeers, C. J. M. van Noesel, D. S. Peeper, M. M. van den Heuvel, K. Schulze

**Affiliations:** 1 Department of Thoracic Oncology, The Netherlands Cancer Institute, Postbus 90203, 1006 BE Amsterdam, The Netherlands; 2 Division of Molecular Oncology & Immunology, The Netherlands Cancer Institute, Amsterdam, The Netherlands; 3 Division of Pathology, The Netherlands Cancer Institute, Amsterdam, The Netherlands; 4 Neogene Therapeutics, Amsterdam, The Netherlands; 5 Core Facility Molecular Pathology & Biobanking, Department of Molecular Pathology, The Netherlands Cancer Institute, Amsterdam, The Netherlands; 6 Department of Pathology, University Medical Centre Groningen, Groningen, The Netherlands; 7 Department of Pathology, OLVG LAB BV, Amsterdam, The Netherlands; 8 Department of Pathology, Academic Medical Center, Amsterdam, The Netherlands; 9 Department of Pulmonology, Radboud University Medical Center, Nijmegen, The Netherlands; 10 Oncology Biomarker Development, Genentech Inc, South San Francisco, USA

**Keywords:** NSCLC, Gene expression, Immunoprofiling, Gene signature

## Abstract

**Background:**

The tumor immune microenvironment is a heterogeneous entity. Gene expression analysis allows us to perform comprehensive immunoprofiling and may assist in dissecting the different components of the immune infiltrate. As gene expression analysis also provides information regarding tumor cells, differences in interactions between the immune system and specific tumor characteristics can also be explored. This study aims to gain further insights in the composition of the tumor immune infiltrate and to correlate these components to histology and overall survival in non-small cell lung cancer (NSCLC).

**Methods:**

Archival tissues from 530 early stage, resected NSCLC patients with annotated tumor and patient characteristics were analyzed using the NanoString nCounter Analysis system.

**Results:**

Unsupervised clustering of the samples was mainly driven by the overall level of inflammation, which was not correlated with survival in this patient set. Adenocarcinoma (AD) showed a significantly higher degree of immune infiltration compared to squamous cell carcinoma (SCC). A 34-gene signature, which did not correlate with the overall level of immune infiltration, was identified and showed an OS benefit in SCC. Strikingly, this benefit was not observed in AD. This difference in OS in SCC specifically was confirmed in two independent NSCLC cohorts. The highest correlation between expression of the 34-gene signature and specific immune cell populations was observed for NK cells, but although a plausible mechanism for NK cell intervention in tumor growth could be established in SCC over AD, this could not be translated back to immunohistochemistry, which showed that NK cell infiltration is scarce irrespective of histology.

**Conclusions:**

These findings suggest that the ability of immune cell infiltration and the interaction between tumor and immune cells may be different between AD and SCC histology and that a subgroup of SCC tumors seems more susceptible to Natural Killer cell recognition and killing, whereas this may not occur in AD tumors. A highly sensitive technique like NanoString was able to detect this subgroup based on a 34-gene signature, but further research will be needed to assist in explaining the biological rationale of such low-level expression signatures.

## Background

In the last decades, it has become increasingly evident that the host immune system has an elaborate interaction with tumor cells. The tumor microenvironment involves a whole range of immune cells together with a wide spectrum of soluble chemokines and cytokines that regulate the infiltrating capacity and the effectiveness of the immune response [1, 2]. The tumor immune microenvironment is a heterogeneous entity, although tumors are often broadly classified as inflamed or ‘hot’ vs. non-inflamed or ‘cold’. Typically, inflamed or ‘hot’ tumors show an abundance of tumor-infiltrating lymphocytes (TILs), IFNγ-producing CD8^+^ T cells and high expression of the inhibitory immune checkpoint programmed death-ligand 1 (PD-L1) suggesting a pre-existing antitumor immune response. In contrast, non-inflamed or ‘cold’ tumors contain hardly any TILs and rarely express PD-L1 [3, 4]. As this is a practical approach, in reality, only a small fraction of tumors seems obviously cold or clearly hot, and the level of inflammation seems more like a spectrum.

Aside from TILs, numerous other immunosuppressive and immunostimulatory mechanisms play a role in the interaction of the immune system with tumor cells. Gene expression analysis allows us to perform comprehensive immunoprofiling and may assist in dissecting the different components of the immune infiltrate. Investigating patterns of the separate components could lead to a better understanding of the complex tumor-immune interaction. This is relevant as presence of inflammatory cells has shown prognostic benefit in non-small cell lung cancer (NSCLC) and other solid tumors probably as representation of the immunostimulatory mechanism at work [5, 6]. On the other hand, myeloid-derived suppressor cells and T regulator cells have an immunosuppressive effect on cytotoxic T cells and have been associated with detrimental effects on the anti-tumor immune response [7]. As gene expression analysis also provides information regarding tumor cells, differences in interactions between the immune system and specific tumor characteristics can also be explored. Ultimately, this knowledge may lead towards a better understanding how the immune composition can be influenced for the patients’ benefit.

This study aims to gain further insights in the composition of the tumor immune infiltrate by nCounter (Nanostring) gene expression analysis and to correlate these components to histology and OS in a large cohort of previously untreated, resected early stage NSCLC samples.

## Methods

### Sample collection and patient cohort

The cohort included 641 formalin-fixed, paraffin-embedded (FFPE) NSCLC samples derived from lung resections performed between 1990 and 2013 at one of four Dutch medical centers. Clinical data about gender, smoking status, neo-adjuvant and adjuvant treatment, age at resection, type of resection, tumor stage, progression free survival (PFS) and overall survival (OS) were collected. No data on treatment after relapse of disease was available. All tumors were histopathologically classified according the 2015 WHO classification system. TNM classification was redefined for resections that were done before 2010 according to the 7th lung cancer TNM classification and staging system [8]. Prior to analysis, the samples were de-identified. The Translational Research Board of the Netherlands Cancer Institute-Antoni van Leeuwenhoek hospital (NKI-AVL) approved the use of patient material in this study.

### Mutation analysis and immunohistochemistry staining

Details on mutational analysis and immunohistochemical (IHC) staining for PD-L1 expression and CD8 infiltration was previously reported [9]. Double staining CD3 (yellow) followed by CD56 (purple) of whole slide sections prepared from FFPE resection specimens was performed on a Discovery Ultra autostainer. Slides were deparaffinised in the instrument and heat-induced antigen retrieval was carried out using Cell Conditioning 1 (CC1, Ventana Medical Systems) for 32 min at 95 °C. The CD3 was detected in the first sequence using clone SP7 (1/100 dilution, 32 min at 37 °C, ThermoScientific). CD3 bound antibody was visualized using Anti-Rabbit NP (Ventana Medical systems) for 12 min at 37 °C followed by Anti-NP AP (Ventana Medical systems) for 12 min at 37 °C, followed by the Discovery Yellow detection kit (Ventana Medical Systems). In the second sequence of the double staining procedure CD56 was detected using clone MRQ-42 (1:2000 dilution, 32 min at 37 °C, Cell Marque). CD56 was visualized using Anti-Rabbit HQ (Ventana Medical systems) for 12 min at 37 °C followed by Anti-HQ HRP (Ventana Medical systems) for 12 min at 37 °C, followed by the Discovery Purple Detection Kit (Ventana Medical Systems). Slides were counterstained with Hematoxylin and Bluing Reagent (Ventana Medical Systems).

### Nanostring analysis

Gene expression analysis was performed using the NanoString nCounter Analysis system (NanoString) on 80–200 ng RNA extracted from FFPE tissue samples. An input of 5*5 μm slides was used. The most tumor-dense area and tumor percentage was assessed by a pathologist on the Hematoxylin and Eosin (H&E) staining and scraped off using a surgical blade. The RNA was isolated using the Roche “High pure RNA paraffin kit” (cat. No. 3270289001) following manufacturers protocol. A customized gene panel (version 0.3), including 531 targets including multiple genes of immunologic function and cancer biology and including 4 housekeeping genes was applied (Additional file 1). For 573 adequate RNA was available for NanoString analysis. To assess the quality of these samples, levels of expression for positive controls and negative controls were retrieved for each sample (Additional file 2). For 18 samples (3.1%) the expression levels were too low and an additional 25 samples (4.4%) failed the NanoString QC, leaving 530 samples for further analysis, consisting of 275 adenocarcinomas (AD), 235 squamous cell carcinomas (SCC) and 20 large cell carcinomas not otherwise specified (NSCLC NOS) (Additional file 3).

### Gene expression and statistical analysis

All data analysis was performed in R (version 3.4.3) using CRAN and Bioconductor packages (Huber, Nature methods 2015). Differential gene expression between AD and SCC was assessed with Limma [10]. Heatmaps were generated with a custom version of ‘heatmap.2’ from the gplots package (https://CRAN.R-project.org/package=gplots). Kaplan–Meier plots were generated using the ‘survival’ package (https://CRAN.R-project.org/package=survival).

### Validation cohorts

Normalized and clinical data were downloaded for two NSCLC datasets (GSE8894 and GSE14814) from NCBI’s GEO database [11, 12]. Z-scores were calculated by centering and scaling the expression data. Expression of the 34-gene signature was computed using the average expression (z-score) of the 34 genes for each sample. To define the ‘34-gene signature high’ and ‘34-gene signature low’ groups for survival analysis the same percentages as in the Nanostring nCounter discovery dataset were used.

RNA sequence read count data of lung squamous cell tumor samples (LUSC) from The Cancer Genome Atlas (TCGA) database were downloaded using TCGAbiolinks [13]. Stage I and II samples that were defined as ‘Primary solid Tumor’ were selected. Statistical analysis of the differential expression of genes was performed using DESeq2 [14].

### Correlation of gene signature to immune cell types

To correlate expression of the 34-gene signature with specific immune cell types Microenvironment Cell Population (MCP)-counter was used [15]. To plot the MCP-counter output samples were ordered according to the expression of the 34-gene signature. Correlations between the 34-gene signature and MCP-counter output was calculated using the ‘Pearson’ correlation.

## Results

### Gene expression analysis

**Fig. 1:**
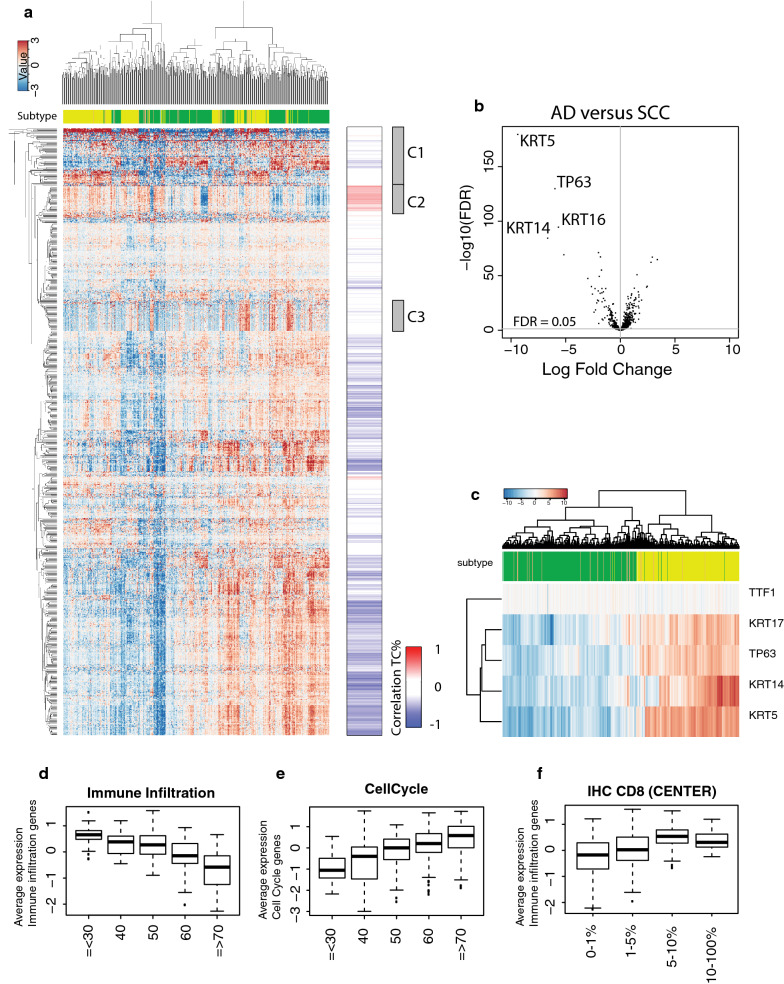
Gene expression patterns in NSCLC. **a** Heatmap and clustering of all NSCLC samples (n = 530) and all genes analyzed using nCounter (NanoString). Top bar indicates the histology as assessed by pathology: green represents AD, yellow SCC and orange NSCLC NOS. Bar right of the heatmap show the correlation of each gene with the percentage of tumor cells (assessment by pathologist). Red indicates a positive correlation, blue a negative correlation. Grey boxes indicate the identified clusters that do not correlate with tumor cell percentages. **b** Volcano plot with the logfold change on the x-axis and FDR (−log10) on the y-axis. The 4 genes with the highest fold change are indicated. **c** Top 4 genes that best differentiate SCC from AD and TTF-1 expression that does not differentiate. Top bar indicates the histology as assessed by pathology: green represents AD, yellow SCC and orange NSCLC NOS. **d** Immune response genes show a negative correlation with the percentage of tumor cells in a sample as assessed by pathology. **e** Cell cycle related genes show a positive correlation with the percentage of tumor cells in a sample as assessed by pathology (cluster 2). **f** Immune response genes show a positive correlation with the percentage of CD8^+^ T cells in a sample as assessed by pathology

In a cohort of 641 NSCLC archival tissue samples adequate RNA could be isolated from 573 samples and these were sent for nCounter (Nanostring) analysis. Gene expression results were obtained for 530 (92.5%) samples. Despite the large range in age of the FFPE blocks, no association was observed between age of the FFPE blocks or hospital of origin with the QC results. All 530 samples were included in an unsupervised clustering analysis (Fig. 1a). Clear differences between the two main histological subtypes AD and SCC were observed (cluster 1). Differential gene expression analysis between AD and SCC showed the largest fold change for KRT5, KRT14, KRT17 and TP63 (Fig. 1b). These genes are known to be highly expressed in SCC and KRT5 and p63 IHC are important markers in diagnostics of lung cancer. Interestingly, TTF1—the most important diagnostic IHC marker for lung AD—was not able to differentiate between histological subtypes on the nCounter platform. Gene expression of TTF1 was higher compared to the negative controls, but at an overall low expression and variance (Fig. 1c), suggesting that protein expression of TTF1 as the most important marker for adenocarcinoma of the lung may not be represented by high RNA levels. These findings show that the NanoString nCounter platform can be used to robustly perform gene expression analysis, even on old FFPE samples (> 20 years).

### Immune infiltration is anti-correlated with cell cycle related genes

Besides differences between histological subtype, the unsupervised clustering of the samples was mainly driven by the overall level of inflammation; the inflamed or ‘hot’ samples vs. the non-inflamed or ‘cold’ samples. The expression of a subset of genes was negatively correlated with genes involved in inflammation (cluster 2). Gene Ontology analysis showed that the genes in cluster 2 were highly enriched for cell cycle related genes (Fig. 1a, Additional file 1). As tumor cells tend to proliferate faster compared to stromal and/or most immune cells, this negative correlation between proliferation represented by cell cycle gene expression and the level of inflammation within samples might suggest a relation with the number of cancer cells and the number of immune cells within that same sample. Indeed, the percentage of tumor cells, based on H&E staining by a pathologist, correlated positively with the expression for cell cycle genes (R = 0.47) and correlated negatively with the expression of immune related genes in our cohort (R = − 0.57, Fig. 1d–e). Apparently, this occurs even though RNA from tumor samples was extracted from tumor-enriched areas designated on the H&E slide by a pathologist in order to increase tumor purity. In addition, these results suggest that not only the number of tumor cells, but also the number of immune cells is represented in the NanoString data and therefore allows for a quantitative measurement of the immune infiltration in these tumor samples. This was confirmed by an increasing expression of immune related genes per increasing number of CD8^+^ T cells in the tumor-enriched areas (Fig. 1f).

### Inflammation according to histological subtype

**Fig. 2:**
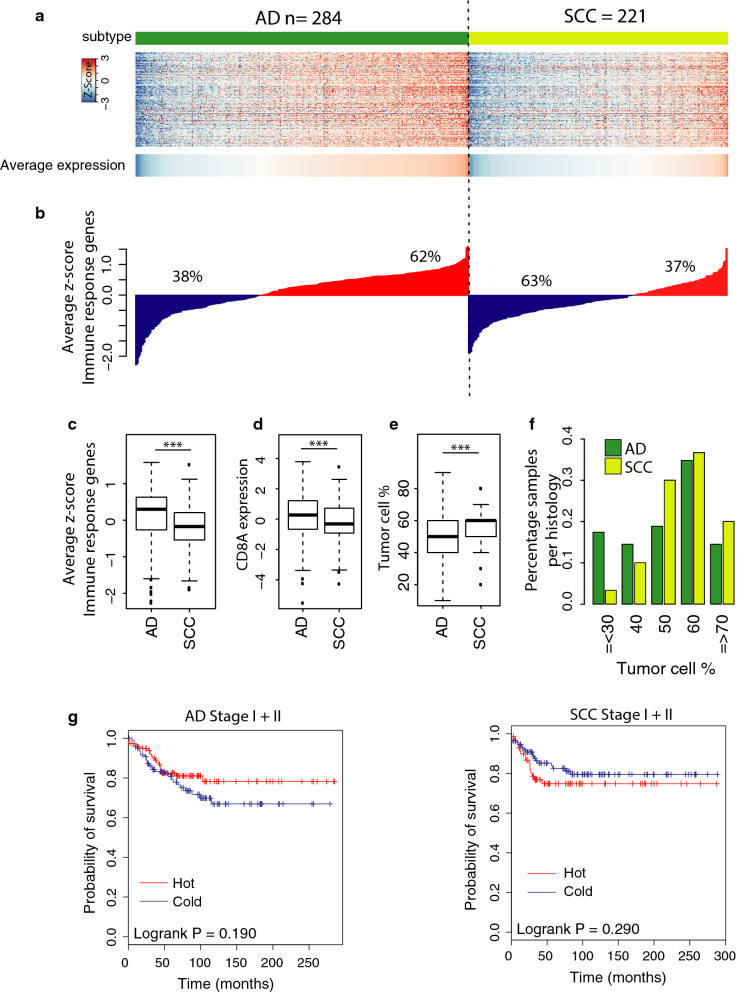
Expression of immune response genes do not provide a survival difference in NSCLC. **a** Heatmap of immune response genes for AD and SCC ordered according to the average expression of the genes. Top bar indicates the histology as assessed by pathology: green represents AD, yellow SCC. **b** Waterfall plot of average expression of immune response related genes, both for AD (left panel) and SCC (right panel). Samples above the average are ‘hot’ tumors (red), the samples below ‘cold’ (blue). **c** Box plot for expression of the immune response related genes per histology. ***p < 0.001. **d** Box plot for expression of *CD8A* per histology. ***p < 0.001. **e** Box plot for mean tumor cell percentages per histology. ***p < 0.001. **f** Bar graph of each tumor cell percentage group for both AD (green) and SCC (yellow) samples. **g** Kaplan–Meier plots with the probability of survival of ‘hot’ versus ‘cold’ tumors in stage I/II tumors, both for AD and SCC

As a proxy to measure the level of ‘active’ inflammation in each sample as opposed to the quantified immune infiltration in general, we calculated the average expression of genes that are known to be involved in the response to immune signals (the ‘immune response genes’ as indicated by NanoString), available in the dataset (Additional file 1). Next, we divided the cohort by histological subtype and tested for each sample whether the average expression of immune response genes was above the mean (‘hot’) or below the mean (‘cold’) of the dataset. The distribution of samples above the mean was 62% for AD versus only 37% for SCC histology (Fig. 2a, b). Based on our previous finding that the level of inflammation is negatively correlated with tumor cell percentage, a comparison between histologies was performed and confirmed our previous result for the ‘immune response gene’ expression as well: tumor cell percentage is significantly higher in SCC (p < 0.001, Fig. 2c, d). These findings suggest that the ability of immune cell infiltration and/or the interaction between tumor and immune cells may be different between AD and SCC histology.

Associations between the level of inflammation and OS benefit has been contradictory for NSCLC in the past. No differences in survival were observed between ‘hot’ and ‘cold’ tumors in stage I/II samples for neither histologies in our cohort (p = 0.19 and 0.29, Fig. 2g).

### Expression of a 34-gene signature is a prognostic marker in SCC

**Fig. 3:**
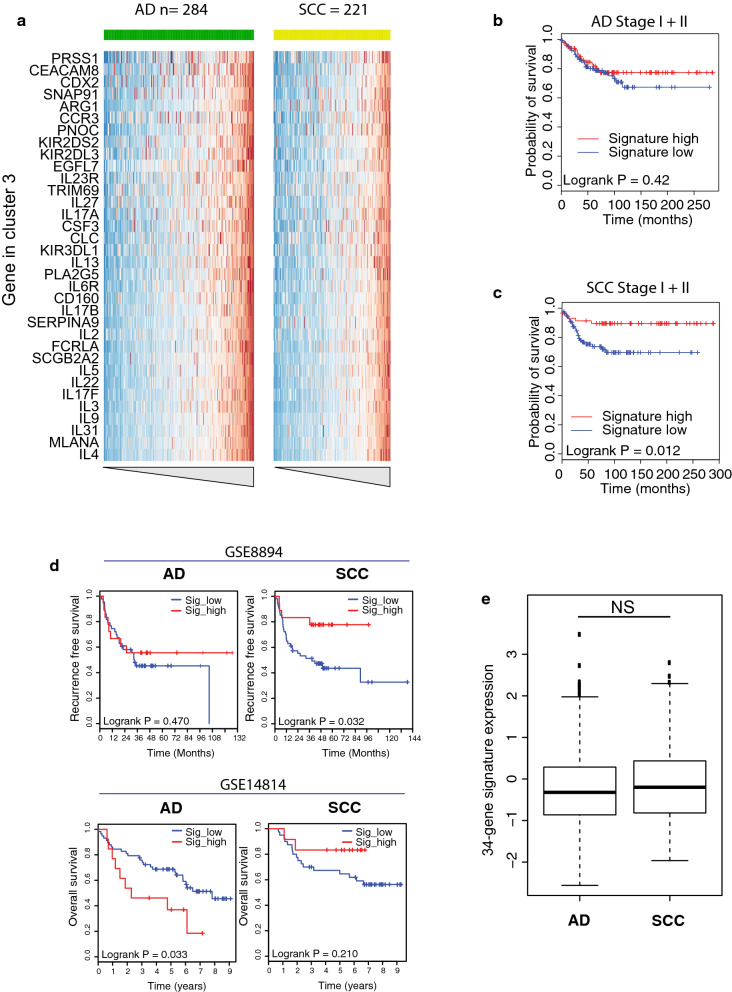
Gene expression cluster 3 is predictive of response in SCC but not in AD. **a** Zoom-in of cluster 3 of the heatmap from Fig. 1a. Samples are ordered on the average expression of the genes per subtype. **b** Kaplan–Meier plots of AD samples divided into high (top 1/3) and low (bottom 2/3) expression of the 34-gene signature. **c** Kaplan–Meier plot of SCC samples divided into high (top 1/3) and low (bottom 2/3) expression of the 34-gene signature. **d** Same analysis as in **b** and **c** in two independent validation sets (GSE8894 and GSE14814). **e** Boxplot of the expression level of the 34-gene signature in AD and SCC samples (p = 0.534)

In addition to the genes that correlated with immune infiltration, histology (cluster 1), and proliferation (cluster 2), the unsupervised clustering of all samples using all genes revealed a third cluster of genes (cluster 3, Figs. 1and 3a). As opposed to the expression of the other immune genes, expression of cluster 3 did not correlate with tumor cell percentage. The expression of the 34-gene signature showed no association with PD-L1 expression and CD8 infiltration (Additional file 4). To check whether there is any clinical relevance in the expression of this set of genes, we performed a survival analysis on the stage I/II samples, both for AD and SCC samples separately. In AD samples, no OS benefit was seen between 34-gene signature high (top 1/3) samples and 34-gene signature low (bottom 2/3) samples (p = 0.42, Fig. 3b). In contrast, a clear OS benefit was observed in SCC between 34-gene signature high (top 1/3) and low (bottom 2/3) samples (p = 0.012, Fig. 3c).

To validate these findings, we downloaded gene expression and associated clinical data from two publicly available NSCLC datasets [11, 12]. Since the expression levels of the genes that comprise the 34-gene signature were generally low, gene expression measured by RNA sequencing failed to provide accurate read count estimates for the 34-gene signature as tested in the TCGA NSCLC dataset (Additional file 2). Therefore, we were confined to methods with a high sensitivity for gene measurement. Microarray data showed similar sensitivity as our nCounter NanoString panel together with positive correlations between the genes of the 34-gene signature (Additional file 2), providing independent datasets to validate our findings.

In concordance with our large cohort of NSCLC samples, survival analysis on a dataset of 61 AD and 72 SCC samples (GSE8894) showed benefit in recurrence free survival (RFS) between samples with high expression (top 1/3) of the 34-gene signature and low expression (bottom 2/3) in SCC (p = 0.032), but not in AD (p = 0.47, Fig. 3d). In the second dataset with 71 AD and 52 SCC samples (GSE14814), survival analysis showed improved OS for the samples with high 34-gene signature expression in SCC albeit not significant (p = 0.21). However, in AD the samples with high expression of the 34-gene signature showed a significant lower OS (p = 0.033, Fig. 3d).

Together, these datasets recurrently show a survival benefit in stage I/II SCC patients with high expression of the identified 34-gene expression signature. This, in contrast to AD patients where high expression of the 34-gene signature is either not or negatively correlated with survival.

### The 34-gene expression signature correlates with NK cell related gene expression

Interestingly, there was no difference in the level of expression of the 34-gene signature between AD and SCC histology (p = 0.53, Fig. 3e). However, high expression of the 34-gene signature was only related to improved survival in SCC, suggests a difference in interaction between tumor and immune cells between the two histological subtypes.

**Fig. 4:**
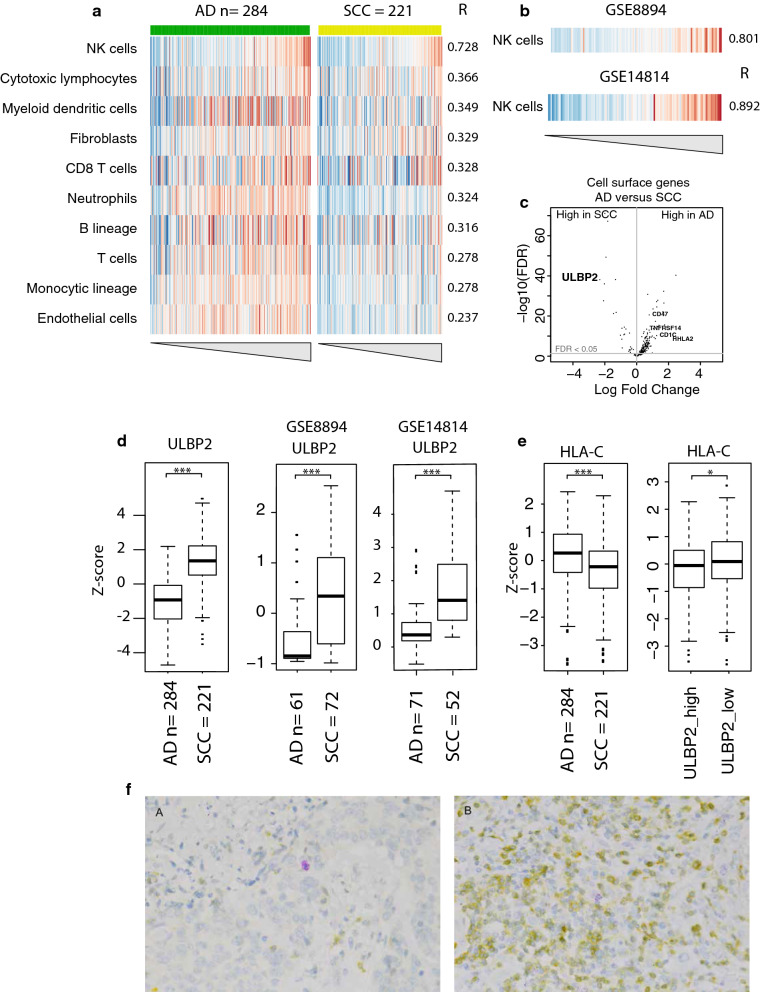
Allocation of the signature. **a** Heatmap of immune cell populations ordered according to expression of the 34-gene signature (cluster 3). **b** Correlation of the NK cell population as measured using MCP-counter in the two independent validation cohorts. Samples are ordered according to the 34-gene expression signature. **c** Volcano plot with the log-fold change on the x-axis and FDR (−log10) on the y-axis in AD and SCC for cell surface genes. **d** Boxplot for expression of *ULBP2* in AD vs. SCC in our dataset and the two independent validation sets. ***p < 0.001. **e** Boxplot for expression of *HLA*-*C* in AD vs. SCC and boxplot with the expression of *HLA*-*C* in *ULBP2* high vs *ULBP2* low samples. *p < 0.05, ***p < 0.001. **f** Examples of a CD56^+^/CD3^−^ NK cell in a 34-gene signature high SCC sample (**a**) and in a 34-gene signature low AD sample (**b**)

To investigate the origin of this beneficial prognostic signal in SCC, we correlated the expression of our 34-gene signature with the presence of specific immune cell populations within the samples. Therefore, we applied MCP-counter on our datasets of 530 samples [15]. The highest correlation between expression of the 34-gene signature and specific immune cell populations was observed for Natural Killer (NK) cells (R = 0.73, Fig. 4a). These finding were corroborated in the two independent datasets with again the highest correlation of the NK cell population (GSE8894, R = 0.80 and GSE14814, R = 0.89, Fig. 4b and Additional file 5).

Although the expression level was comparable between histologies, but high expression of the 34-gene signature was only related to improved survival in SCC, this may suggest a difference in interaction between tumor and immune cells between the two histological subtypes. To further test whether the improved survival in SCC, but not in AD, even though expression level of the 34-gene signature was similar in both histologies, could indeed be explained by differences in the interface between tumor cells and immune microenvironment between both histologies, we analyzed the dataset for cell surface genes and compared their expression between AD and SCC samples (Fig. 4c). Interestingly, one of the cell surface genes highly expressed in SCC but not in AD is *ULBP2* (FDR < 0.001, Fig. 4d), a marker for NK cell killing. Higher expression of *ULBP2* in SCC was also observed in our validation datasets (GSE8894; FDR < 0.001 and GSE14814; FDR < 0.001, Fig. 4d). Also, high expression of *ULBP2* was associated with lower expression of *HLA*-*C*, one of the genes encoding for major histocompatibility complex (MHC) class I molecules. Furthermore, expression of *HLA*-*C* was significantly lower in SCC compared to AD (Fig. 4e).

To further explore the possible role of NK cell killing in regard to the OS benefit in signature-high SCC opposed to signature-high AD, a double-staining of CD56 and CD3 was performed in a selection of samples. Signature-high and signature-low in both AD and SCC samples were evaluated. Overall, the infiltration of CD56^+^/CD3^−^ cells was scarce in SCC and only somewhat more frequent in AD, both irrespective of the expression level of the 34-gene signature. This difference between AD and SCC presumably matches the previously mentioned difference in tumor cell percentage and amount of immune infiltrate between histologies, which is overall more pronounced in AD vs. SCC (Fig. 4f). These findings might suggest that a subgroup of SCC tumors seems more susceptible to NK cell recognition and killing, whereas this may not occur in AD tumors.

## Discussion

In our study, we performed gene expression analysis on a large cohort of early stage resected NSCLC samples. Unsupervised clustering of the samples was mainly driven by the overall level of inflammation, which was not correlated with survival in this patient set. Expression of a 34-gene signature did not correlate with the general inflammation level. This signature provided an OS benefit in SCC, but not in AD. This finding was validated in two independent NSCLC cohorts. The signature showed the strongest association with NK cells based on gene expression profiling, but this could not be validated by IHC, which showed that NK cell infiltration is scarce irrespective of histology.

The expression level of the 34-gene signature was comparable in both histological subtypes, but had a different effect on OS. This histology-dependent OS benefit may suggest a difference in the interaction of the immune system between AD and SCC NSCLC. To understand the biological foundation of the 34-gene signature, the selection of genes in the signature was compared to the gene profiles of eight immune cell populations as established by the MCP-counter method [15]. Our gene signature showed the strongest correlation with the gene profile of NK cells. NK cells have the unique property to revert to cell-killing induced without presentation of tumor specific antigens [16]. Production and release of granules, like perforin and granzyme B, cause lyses of the targeted cell [17]. Inhibition of NK cells occurs through activation of killer cell immunoglobulin-like receptors (KIRs) by recognition of MHC class I molecules on surrounding cells and thereby providing protection against auto-immunity. One mechanism of tumor immune escape is downregulation of MHC class I on tumor cells in order to evade T cell recognition and killing [18]. However, this may render them vulnerable to NK cell attack. To strengthen the rationale for annotating our signature as possessing NK cell features, we sought for differences between the two histological subtypes in expression of tumor-related genes (as opposed to immune-related genes for which our NanoString panel was enriched). In our cohort, SCC samples showed a significant higher expression of the NK activation marker *ULBP2* and lower expression of the MHC class I gene *HLA*-*C* compared to AD samples. This may suggest that tumor growth in SCC may be possible because of the tumor immune escape mechanism of evasions of T cell recognition, but that NK cell killing may successfully prevent this escape, eventually leading to improved OS. McGranahan et al. recently found that loss of heterozygosity of HLA (HLA LOH) seemed to be correlated to prior immune activation and to a higher mutational burden in treatment-naïve, resected NSCLC [19]. Even though McGranahan et al. also found a higher overall level of inflammation in AD compared to SCC samples, SCC more often showed HLA LOH and this was associated with a higher expression of two different NK cell signatures from RNA sequencing data.

Unfortunately, there is no clearly validated method for establishing NK cell infiltration by IHC [20]. Because NK cells were defined as CD56^+^/CD3^−^ in the MCP-counter method, we performed a double-staining with CD56 and CD3 on a selection of samples in this cohort, but very few infiltrating NK cells in either histology were seen [15]. It has been described that even at a low ratio NK cells are able to kill tumor cells due to their specific cytotoxic abilities [21]. As the presence of NK cells in the tumor microenvironment is scarce, it may be difficult to study the role of the innate immune system and NK cells in particular regarding tumor cell attack [22, 23]. Furthermore, by performing only a double-staining with CD56 and CD3 acquiring a differentiating signal from additional subtypes of NK cells could have been missed; nor is it possible to establish the activity-level of these specific NK cells. Infiltration of tumors by NK cells has been previously linked to favorable outcome, although there are limited studies performed in NSCLC [24]. Villegas et al. found improved OS in early-stage SCC NSCLC when more NK cells were present in the tumor as assessed by CD57 staining [25].

However, the NanoString nCounter system used in this study and the microarray-based techniques used in both validation cohorts provide a higher sensitivity compared to standard RNA sequencing. This technique therefor allows discovery of immune gene expression that is present in very low abundance within the tumor microenvironment. Indeed, expression of most genes in the 34-gene signature was low, which precludes accurate measurement of the 34-gene signature in RNA sequencing data sets like TCGA and therefor precludes validation of the prognostic ability of the signature in these available cohorts. Backman et al. found no correlation between IHC of the NK cell marker NKp46 and expression of the corresponding gene *NCR1* measured by RNA sequencing in early-stage NSCLC, which they ascribed to low abundance of NK cells as well [26]. They also noticed that the expression of NK cell genes was not associated with the overall level of inflammation. This NK-enriched subgroup had low expression of T cell markers, low T cell activation and a low tumor mutational burden. Interestingly, the prognosis of this subgroup was similar to the inflamed subgroup, suggesting that not neoantigen-driven T cell recruitment, but a different (immune) mechanism of containing tumor growth may be responsible. Unlike our findings, this OS benefit was irrespective of histology.

Unfortunately, we were unable to provide solid evidence for the annotation of the 34-signature. The signature seemed to have NK cell like features, but although a plausible mechanism for NK cell intervention in tumor growth could be established in SCC over AD, this could not be translated back to IHC or RNA sequencing data. Unfortunately, exploration of additional pathways or gene sets associated with the 34-gene signature was not possible due to the relatively small number of genes in our NanoString panel, which was highly enriched for immune genes specifically, and no additional RNA sequencing data of this cohort was available. Previous NK cell signatures were based on RNA sequencing, sorted cell or single cell RNA sequencing. Due to the low expression level of most genes in the 34-gene signature a formal comparison between signatures that use different techniques seems futile. Maybe single cell sequencing using NanoString or microarray-based techniques may solve the remaining questions regarding the underlying mechanisms of scarce immune cells in the tumor microenvironment.

## Conclusion

In conclusion, this study identified a subgroup of squamous NSCLC with an OS benefit that seemed not related to infiltration of immune cells in general, suggesting that a different (immune) mechanism of containing tumor growth may be responsible. A highly sensitive technique like NanoString was able to detect this subgroup based on a 34-gene signature, but further research will be needed to assist in explaining the biological rationale of such low-level expression signatures.

## Supplementary information

**Additional file 1.** NanoString gene panel.

**Additional file 2: Figure S1.** A) Flowchart of samples for NanoString analysis. B) QC data NanoString: positive/negative controls and keratin expression. C) The 34-gene signature does not work on TCGA RNA-seq data: unmeasurable or low expression of the majority of the genes. D) Heatmap with correlations (Pearson correlation) of genes from the 34-gene signature in the NSCLC validation set (GSE14814).

**Additional file 3.** Patients’ and tumor characteristics of the non-small cell lung cancer cohort.

**Additional file 4: Figure S2.** Boxplots of the associations between the immune response genes and the 34-gene signature with PD-L1 expression on tumor cells (TC) and immune cells (IC) and CD8 infiltration in the tumor margin and in the tumor center.

**Additional file 5: Figure S3.** Heatmaps of the cluster 3 genes with allocated immune cell types per two independent cohort with correlation.

## Data Availability

The datasets used and/or analyzed during the current study are available from the corresponding author on reasonable request.

## Abbreviations

TIL: Tumor-infiltrating lymphocytes
PD-L1: Programmed death-ligand 1
NSCLC: Non-small cell lung cancer
FFPE: Formalin-fixed, paraffin-embedded
PFS: Progression free survival
OS: Overall survival
NKI-AVL: Netherlands Cancer Institute-Antoni van Leeuwenhoek hospital
IHC: Immunohistochemical
H&E: Hematoxylin and Eosin
AD: Adenocarcinoma
SCC: Squamous cell carcinoma
NSCLC NOS: Large cell carcinomas not otherwise specified
TCGA: The Cancer Genome Atlas
LUSC: Lung squamous cell tumor sample
MCP: Microenvironment Cell Population
RFS: Recurrence free survival
NK: Natural Killer (NK)
MHC: Major histocompatibility complex
KIR: Killer cell immunoglobulin-like receptor

## References

[1] Vesely MD, Kershaw MH, Schreiber RD, Smyth MJ (2011). Natural innate and adaptive immunity to cancer. Annu Rev Immunol.

[2] Fridman WH, Pages F, Sautes-Fridman C, Galon J (2012). The immune contexture in human tumours: impact on clinical outcome. Nat Rev Cancer.

[3] Herbst RS, Soria JC, Kowanetz M, Fine GD, Hamid O, Gordon MS (2014). Predictive correlates of response to the anti-PD-L1 antibody MPDL3280A in cancer patients. Nature.

[4] Hegde PS, Karanikas V, Evers S (2016). The where, the when, and the how of immune monitoring for cancer immunotherapies in the era of checkpoint inhibition. Clin Cancer Res.

[5] Kayser G, Schulte-Uentrop L, Sienel W, Werner M, Fisch P, Passlick B (2012). Stromal CD4/CD25 positive T-cells are a strong and independent prognostic factor in non-small cell lung cancer patients, especially with adenocarcinomas. Lung Cancer.

[6] Bremnes RM, Busund LT, Kilvaer TL, Andersen S, Richardsen E, Paulsen EE (2016). The role of tumor-infiltrating lymphocytes in development, progression, and prognosis of non-small cell lung cancer. J Thorac Oncol.

[7] O’Donnell JS, Teng MWL, Smyth MJ (2019). Cancer immunoediting and resistance to T cell-based immunotherapy. Nat Rev Clin Oncol..

[8] Goldstraw P, Crowley J, Chansky K, Giroux DJ, Groome PA, Rami-Porta R (2007). The IASLC Lung Cancer Staging Project: proposals for the revision of the TNM stage groupings in the forthcoming (seventh) edition of the TNM classification of malignant tumours. J Thorac Oncol.

[9] Theelen W, Kuilman T, Schulze K, Zou W, Krijgsman O, Peters D (2019). Absence of PD-L1 expression on tumor cells in the context of an activated immune infiltrate may indicate impaired IFNgamma signaling in non-small cell lung cancer. PLoS ONE.

[10] Ritchie ME, Phipson B, Wu D, Hu Y, Law CW, Shi W (2015). limma powers differential expression analyses for RNA-sequencing and microarray studies. Nucleic Acids Res.

[11] Lee ES, Son DS, Kim SH, Lee J, Jo J, Han J (2008). Prediction of recurrence-free survival in postoperative non-small cell lung cancer patients by using an integrated model of clinical information and gene expression. Clin Cancer Res.

[12] Zhu CQ, Ding K, Strumpf D, Weir BA, Meyerson M, Pennell N (2010). Prognostic and predictive gene signature for adjuvant chemotherapy in resected non-small-cell lung cancer. J Clin Oncol.

[13] Colaprico A, Silva TC, Olsen C, Garofano L, Cava C, Garolini D (2016). TCGAbiolinks: an R/Bioconductor package for integrative analysis of TCGA data. Nucleic Acids Res.

[14] Love MI, Huber W, Anders S (2014). Moderated estimation of fold change and dispersion for RNA-seq data with DESeq2. Genome Biol.

[15] Becht E, Giraldo NA, Lacroix L, Buttard B, Elarouci N, Petitprez F (2016). Estimating the population abundance of tissue-infiltrating immune and stromal cell populations using gene expression. Genome Biol.

[16] Trinchieri G (1989). Biology of natural killer cells. Adv Immunol.

[17] Carotta S, Targeting NK (2016). Cells for anticancer immunotherapy: clinical and preclinical approaches. Front Immunol..

[18] Chen DS, Mellman I (2013). Oncology meets immunology: the cancer-immunity cycle. Immunity.

[19] McGranahan N, Rosenthal R, Hiley CT, Rowan AJ, Watkins TBK, Wilson GA (2017). Allele-specific HLA loss and immune escape in lung cancer evolution. Cell.

[20] Cursons J, Souza-Fonseca-Guimaraes F, Foroutan M, Anderson A, Hollande F, Hediyeh-Zadeh S (2019). A gene signature predicting natural killer cell infiltration and improved survival in melanoma patients. Cancer Immunol Res.

[21] Huntington ND, Vosshenrich CA, Di Santo JP (2007). Developmental pathways that generate natural-killer-cell diversity in mice and humans. Nat Rev Immunol.

[22] Lavin Y, Kobayashi S, Leader A, Amir ED, Elefant N, Bigenwald C (2017). Innate immune landscape in early lung adenocarcinoma by paired single-cell analyses. Cell.

[23] Barry KC, Hsu J, Broz ML, Cueto FJ, Binnewies M, Combes AJ (2018). A natural killer-dendritic cell axis defines checkpoint therapy-responsive tumor microenvironments. Nat Med.

[24] Tuminello S, Veluswamy R, Lieberman-Cribbin W, Gnjatic S, Petralia F, Wang P (2019). Prognostic value of immune cells in the tumor microenvironment of early-stage lung cancer: a meta-analysis. Oncotarget..

[25] Villegas FR, Coca S, Villarrubia VG, Jimenez R, Chillon MJ, Jareno J (2002). Prognostic significance of tumor infiltrating natural killer cells subset CD57 in patients with squamous cell lung cancer. Lung Cancer.

[26] Backman M, La Fleur L, Kurppa P, Djureinovic D, Elfving H, Brunnstrom H, et al. Characterization of patterns of immune cell infiltration in non-small cell lung cancer (NSCLC). J Thorac Oncol. 2020. 10.1016/j.jtho.2019.12.127 32028050

