# Supplementary figures and images for: Presence of a 34-gene signature is a favorable prognostic marker in squamous non-small cell lung carcinoma
Source: J Transl Med. 2020 Jul 3;18:271. doi: 10.1186/s12967-020-02436-3 (PMC7333331; doi:10.1186/s12967-020-02436-3)

Supplementary Figure 1

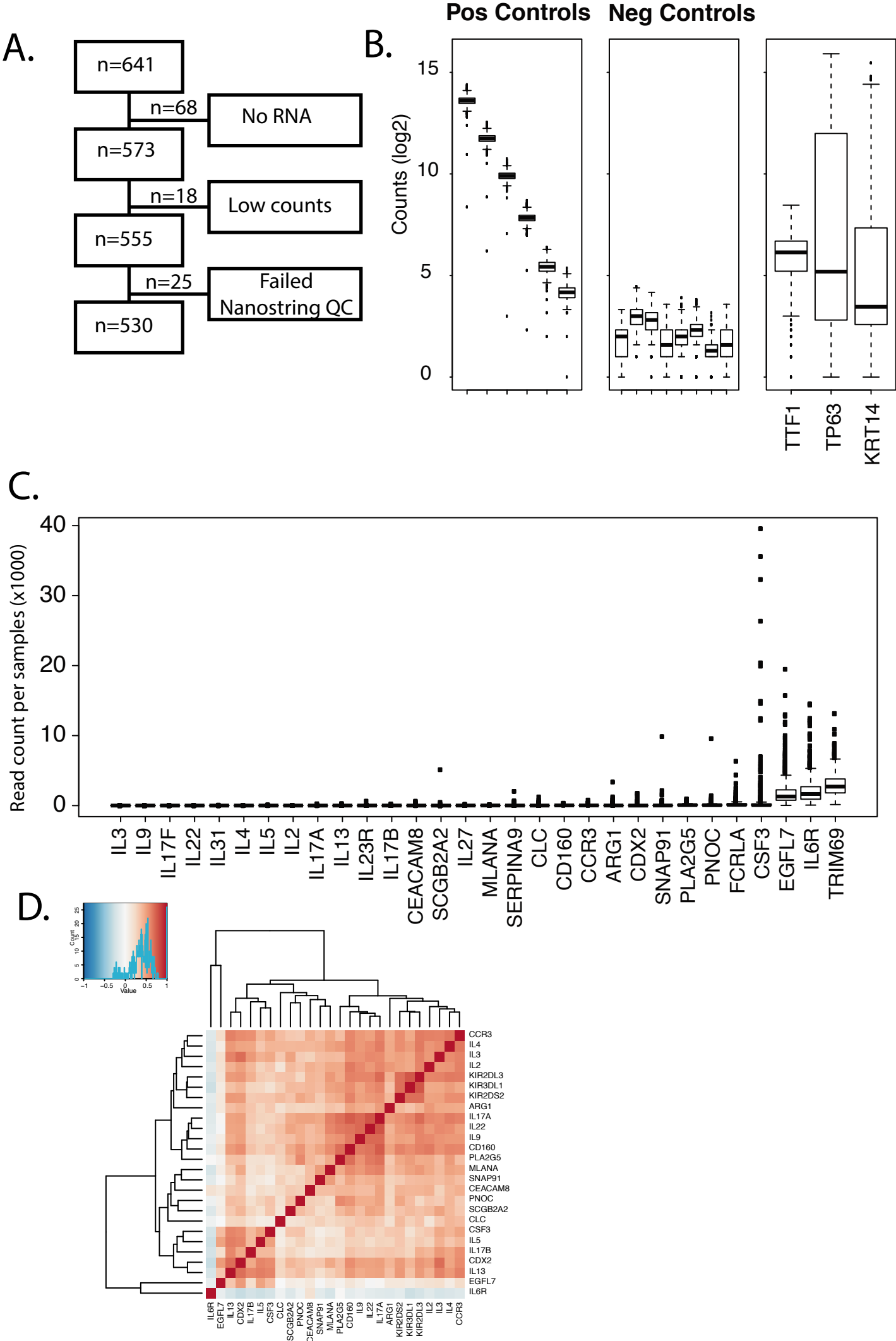

Supplement: Supplementary file 2 — Additional file 2: Figure S1. A) Flowchart of samples for NanoString analysis. B) QC data NanoString: positive/negative controls and keratin expression. C) The 34-gene signature does not work on TCGA RNA-seq data: unmeasurable or low expression of the majority of the genes. D) Heatmap with correlations (Pearson correlation) of genes from the 34-gene signature in the NSCLC validation set (GSE14814). [file 12967_2020_2436_MOESM2_ESM.pdf]

# Supplementary Figure 2

Immune response genes

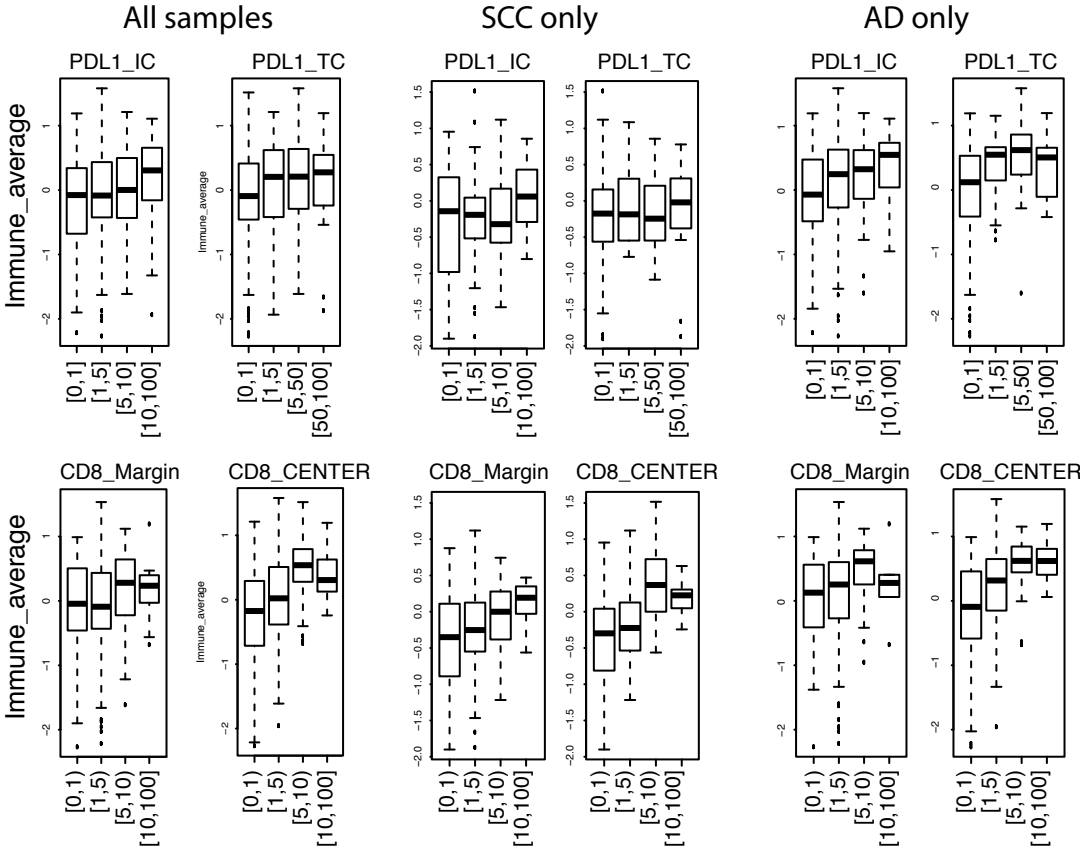

34-gene signature

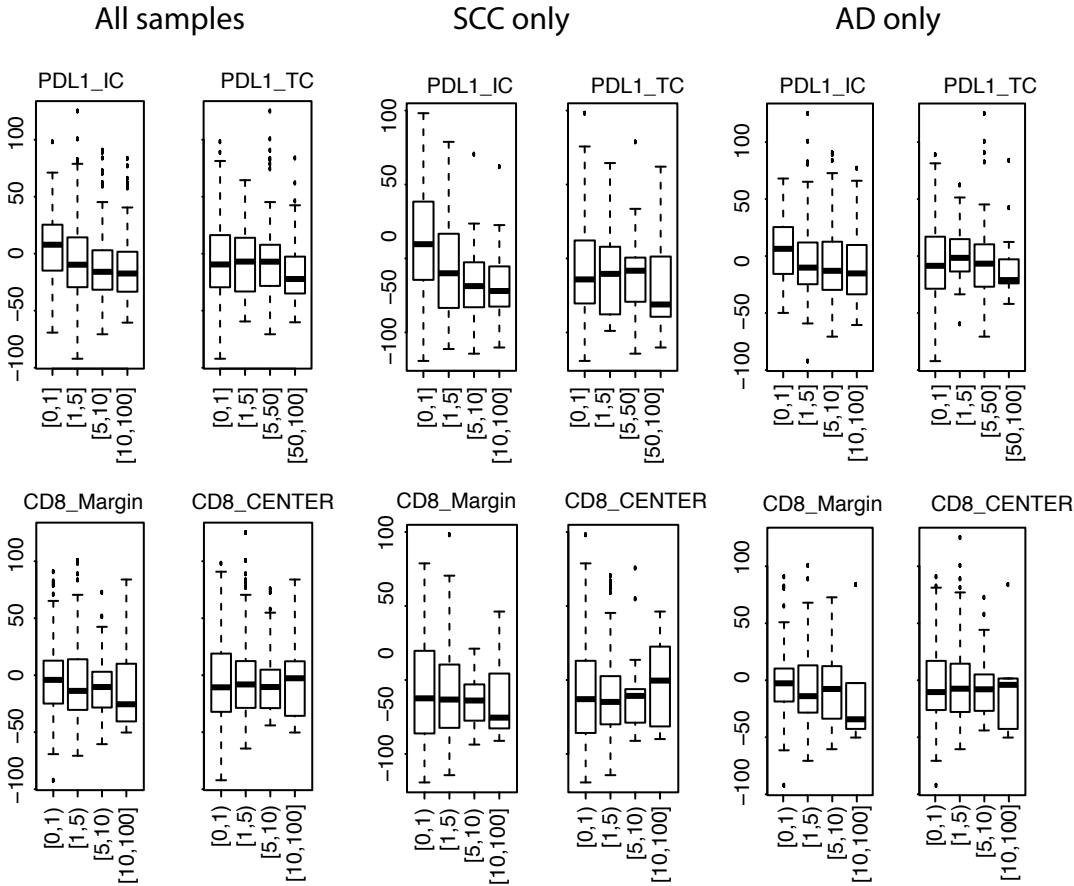

Supplement: Supplementary file 4 — Additional file 4: Figure S2. Boxplots of the associations between the immune response genes and the 34-gene signature with PD-L1 expression on tumor cells (TC) and immune cells (IC) and CD8 infiltration in the tumor margin and in the tumor center. [file 12967_2020_2436_MOESM4_ESM.pdf]

# Supplementary Figure 3

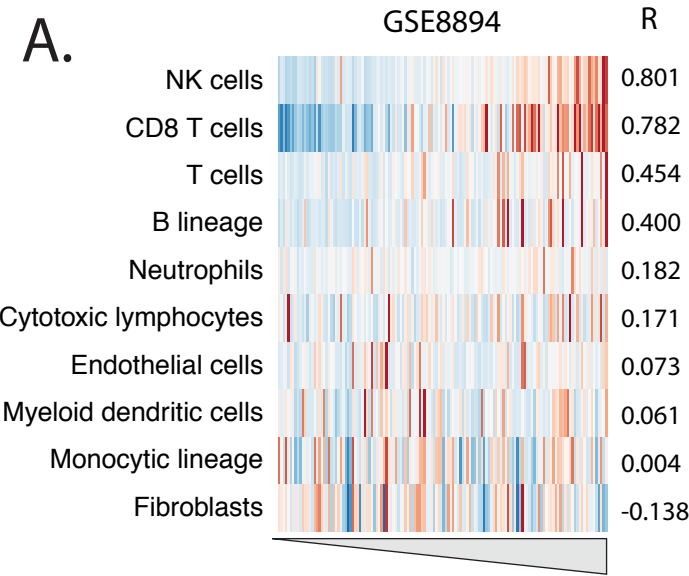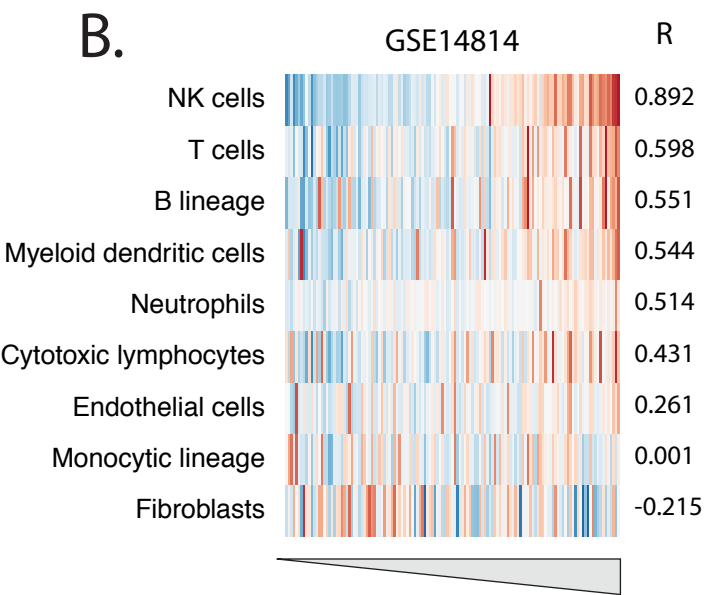

Supplement: Supplementary file 5 — Additional file 5: Figure S3. Heatmaps of the cluster 3 genes with allocated immune cell types per two independent cohort with correlation. [file 12967_2020_2436_MOESM5_ESM.pdf]
