# Supplementary material for: Presence of a 34-gene signature is a favorable prognostic marker in squamous non-small cell lung carcinoma
Source: J Transl Med. 2020 Jul 3;18:271. doi: 10.1186/s12967-020-02436-3 (PMC7333331; doi:10.1186/s12967-020-02436-3)
Supplement: Supplementary file 3 — Additional file 3. Patients’ and tumor characteristics of the non-small cell lung cancer cohort. [file 12967_2020_2436_MOESM3_ESM.docx]

**Additional file 3. Patients’ and tumor characteristics of the non-small cell lung cancer cohort**

* percentages for analyzed samples only. EGFR mutations included exon 19 deletions, exon 20 insertions and exon 21 L858R mutations. No T790M mutations were found. KRAS mutations included mutations in codon 12, 13 and 61. Mutations in AKT1, ERBB2, FLT3, JAK2, KIT, MYD88 were not present within this cohort. All present MET mutations were germline single nucleotide polymorphisms.

SCC = squamous cell carcinoma, AD = adenocarcinoma, NSCLC NOS = non-small cell lung cancer not otherwise specified.
